# Supplementary material for: Activation of the essential kinase PDK1 by phosphoinositide-driven trans-autophosphorylation
Source: Nat Commun. 2022 Apr 6;13:1874. doi: 10.1038/s41467-022-29368-4 (PMC8986801; doi:10.1038/s41467-022-29368-4)
Supplement: Supplementary file 1 — Supplementary Information [file 41467_2022_29368_MOESM1_ESM.pdf]

Activation of the essential kinase PDK1 by phosphoinositide-driven trans-autophosphorylation

Aleksandra Levina[1,2], Kaelin D. Fleming[3], John E. Burke[3,4] and Thomas A. Leonard[1,2,5]

[1]Department of Structural and Computational Biology, Max Perutz Labs, Campus Vienna Biocenter 5, 1030 Vienna, Austria

[2]Department of Medical Biochemistry, Medical University of Vienna, 1090 Vienna, Austria

[3]Department of Biochemistry and Microbiology, University of Victoria, Victoria, BC, Canada V8W 2Y2

[4]Department of Biochemistry and Molecular Biology, The University of British Columbia, Vancouver, British Columbia V6T 1Z3, Canada

[5]Correspondence: thomas.leonard@meduniwien.ac.at

Supplementary Fig. 1. Intact mass spectra of recombinant proteins used in this study.

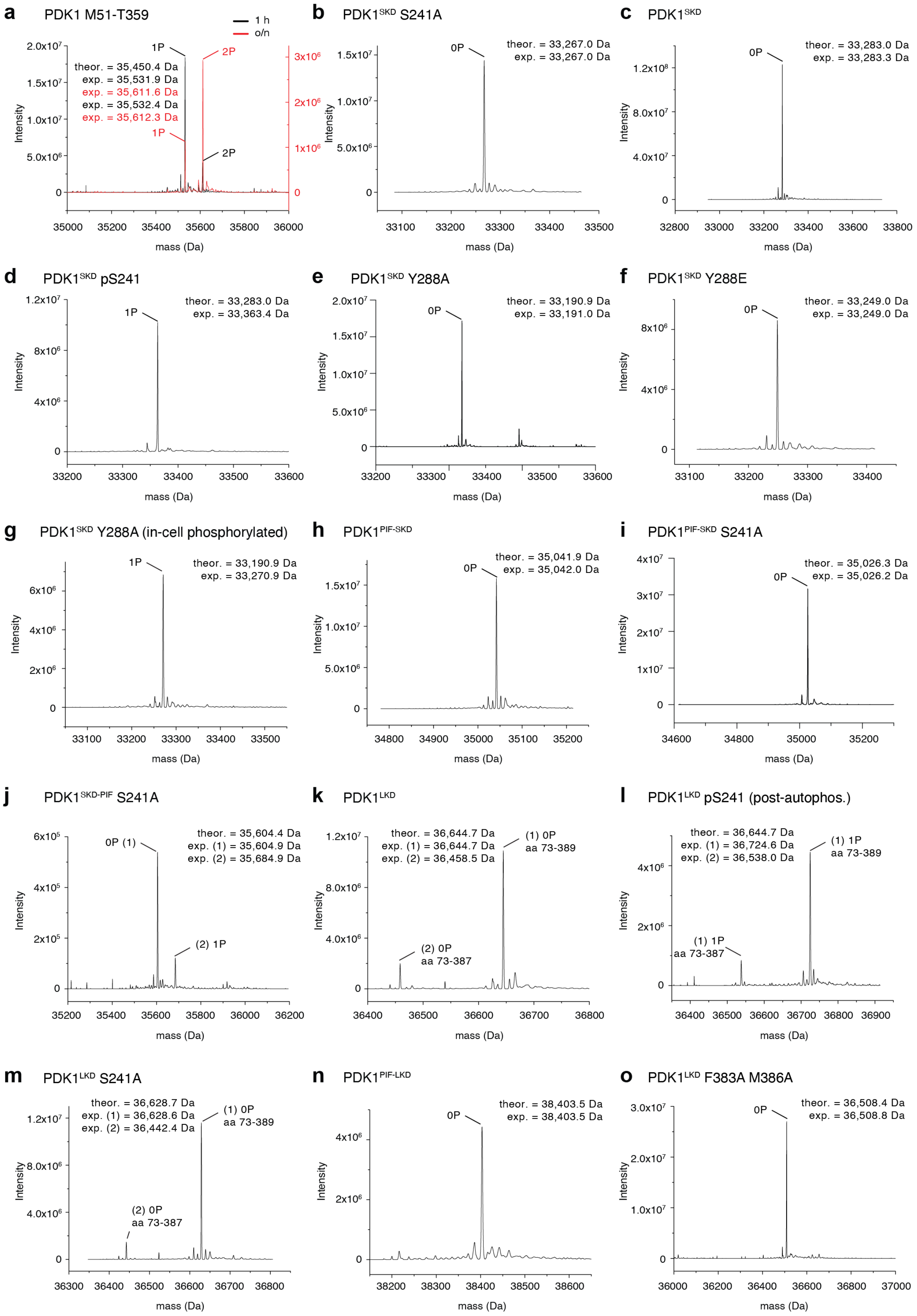

Supplementary Fig. 1. Intact mass spectra of recombinant proteins used in this study.

(A) – (O) Intact mass spectra for constructs used in this study. Theoretical (theor.) and experimentally determined (exp.) masses are reported, as well as any post-translational modifications.

Supplementary Fig. 2. Intact mass spectra of recombinant proteins used in this study.

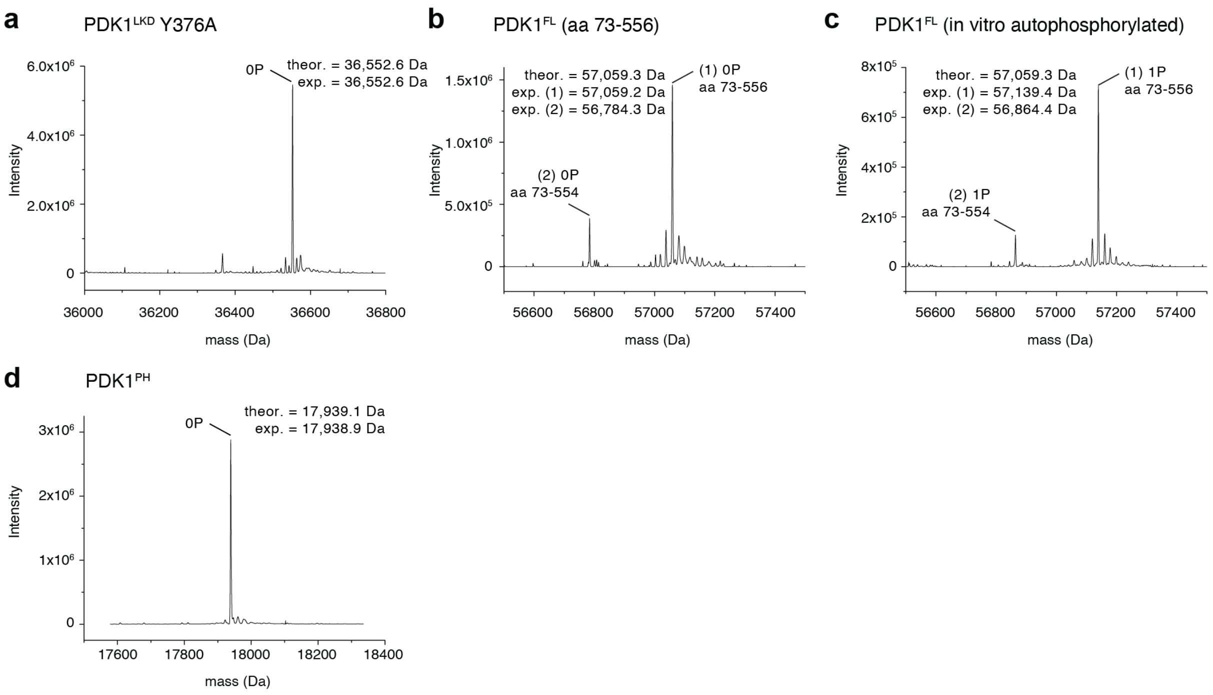

Supplementary Fig. 2. Intact mass spectra of recombinant proteins used in this study.

(A) – (D) Intact mass spectra for constructs used in this study. Theoretical (theor.) and experimentally determined (exp.) masses are reported, as well as any post-translational modifications.

Supplementary Fig. 3. *In silico* modeling of the PDK1 kinase domain dimer.

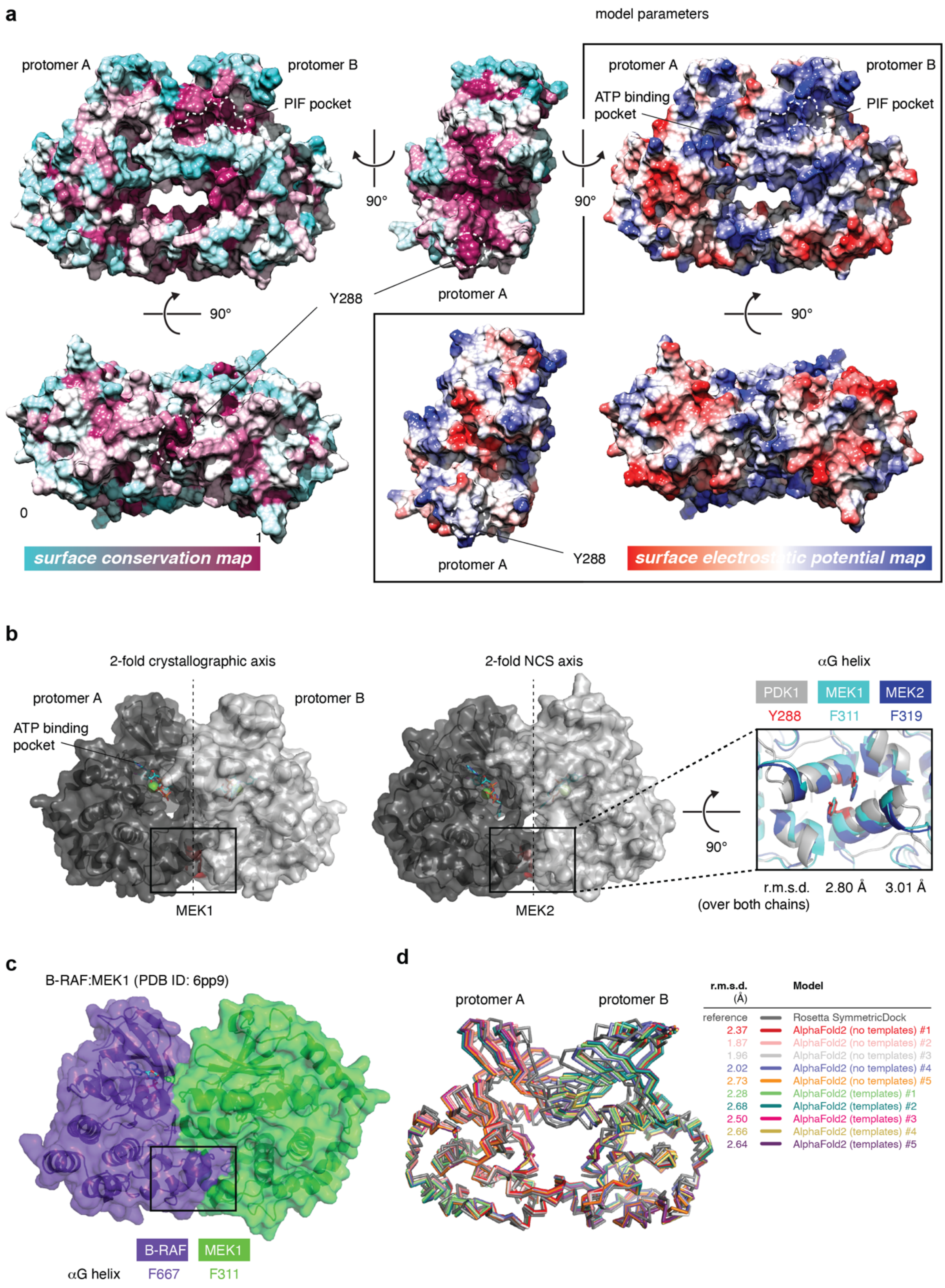

Supplementary Fig. 3. *In silico* modeling of the PDK1 kinase domain dimer.

(A) Surface conservation and surface electrostatic potential mapping onto the PDK1 kinase domain dimer model (Rosetta).

(B) Structures of MEK1 (PDB: 1s9j) and MEK2 (PDB: 1s9i) illustrating crystallographic and non-crystallographic dimers respectively. Inset: view of αG-mediated dimer interface from superposition of PDK1 kinase domain dimer model with MEK1 and MEK2 homodimers. R.m.s.d. values are over all C_{α} atoms for both chains.

(C) B-Raf and MEK1 heterodimer structure (PDB: 6pp9) with annotation of F667 (B-Raf) and F311 (MEK1) in helix αG that are equivalent to Y288 in PDK1.

(D) Comparison of AlphaFold2 predictions of the homodimeric assembly of the PDK1 kinase domain (residues Q73-T359) to the model generated by Rosetta SymmDock. AlphaFold2 was run with and without template matching, and the ten resulting models (five from each run) superimposed on the reference Rosetta model over all Cα atoms.

Supplementary Fig. 4. *In silico* modeling of the PDK1 kinase domain dimer.

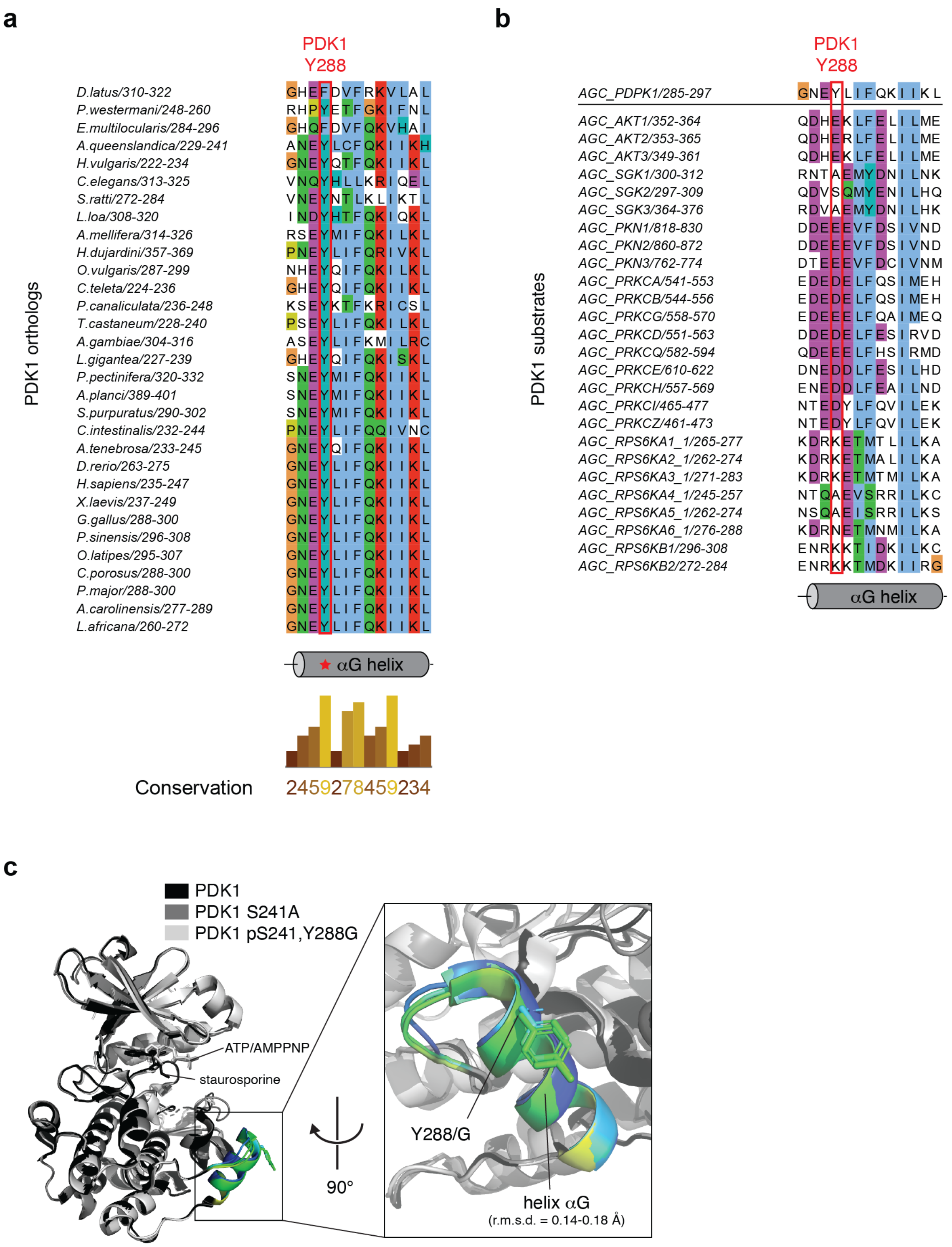

Supplementary Fig. 4. *In silico* modeling of the PDK1 kinase domain dimer.

(A) Sequence alignment of the αG helix in PDK1 orthologs spanning more than 900My of evolution.

(B) Sequence alignment of the αG helix in 26 reported or putative PDK1 substrates (all human sequences).

(C) Crystal structure of PDK1 Y288G (residues 51-359) (PDB : 3hrc). Inset: superposition of wild-type PDK1 (PDB: 2biy) and PDK1 Y288G structures.

Supplementary Fig. 5. A hydrophobic motif in PDK1 drives *trans*-autophosphorylation.

a

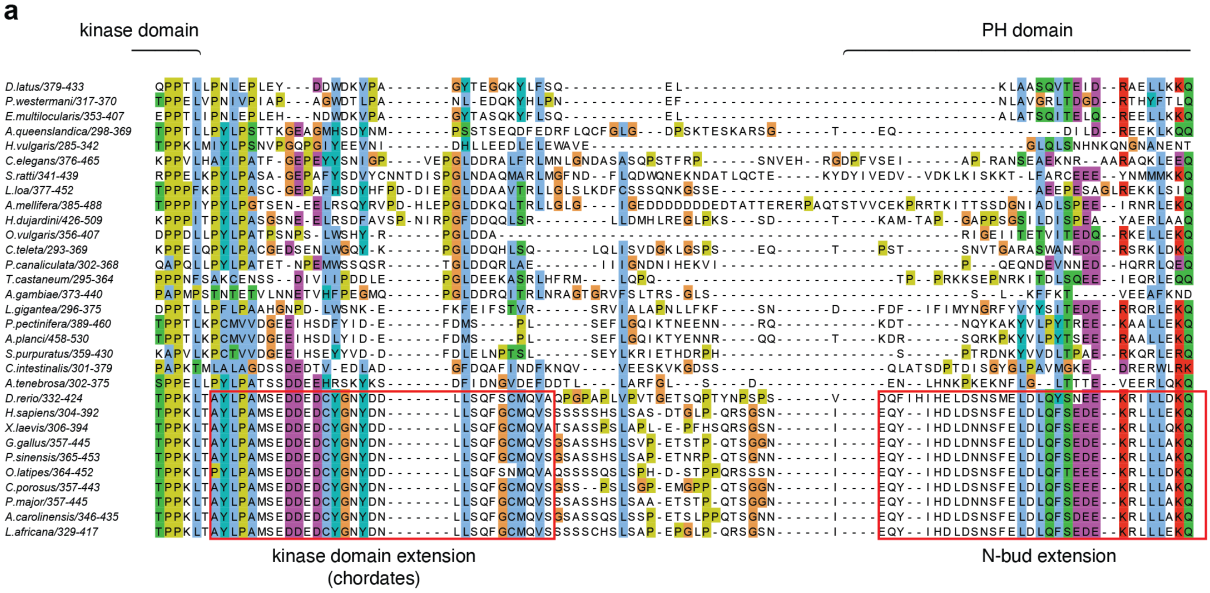

b

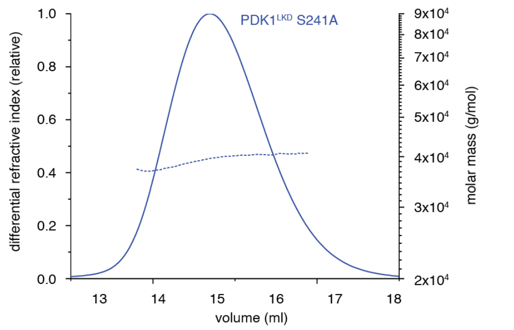

Protein	ATP	Polydispersity	Mr_{exp} (kDa)	Mr_{theor} (kDa)	Mr_{exp}/Mr_{theor}
PDK1[LKD] S241A	+	1.001	39.4	38.8	1.02

c

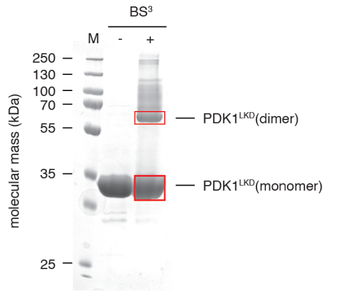

d

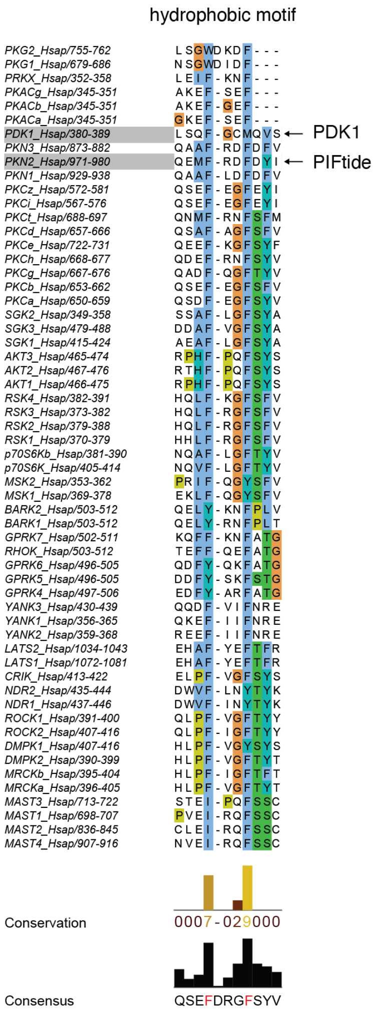

Supplementary Fig. 5. A hydrophobic motif in PDK1 drives trans-autophosphorylation.

(A) Alignment of the kinase-PH interdomain linker of PDK1 isoforms from distantly related species. The conserved kinase domain extension and the N-bud part of the PH domain are highlighted with red boxes.

(B) Size exclusion chromatography coupled to multi-angle light scattering (SEC-MALS) of $PDK1^{LKD}$ S241A in the presence of 1 mM ATP. The table summarizes protein polydispersity, theoretical (Mr_{theor}) and experimentally determined (Mr_{exp}) molecular weights, as well as the calculated oligomeric state (Mr_{exp}/Mr_{theor}).

(C) SDS-PAGE analysis of BS^3-crosslinked $PDK1^{LKD}$ S241A in the presence of 1 mM ATP. Monomer and dimer bands corresponding to the red boxes were excised from the gel and subjected to analysis by mass spectrometry.

(D) Alignment of the hydrophobic motif sequence of human PDK1 identified in this study with the hydrophobic motifs of 58 other AGC kinases. Conservation and consensus sequences are indicated below the alignment.

Supplementary Fig. 6. PDK1 is autoinhibited by its PH domain.

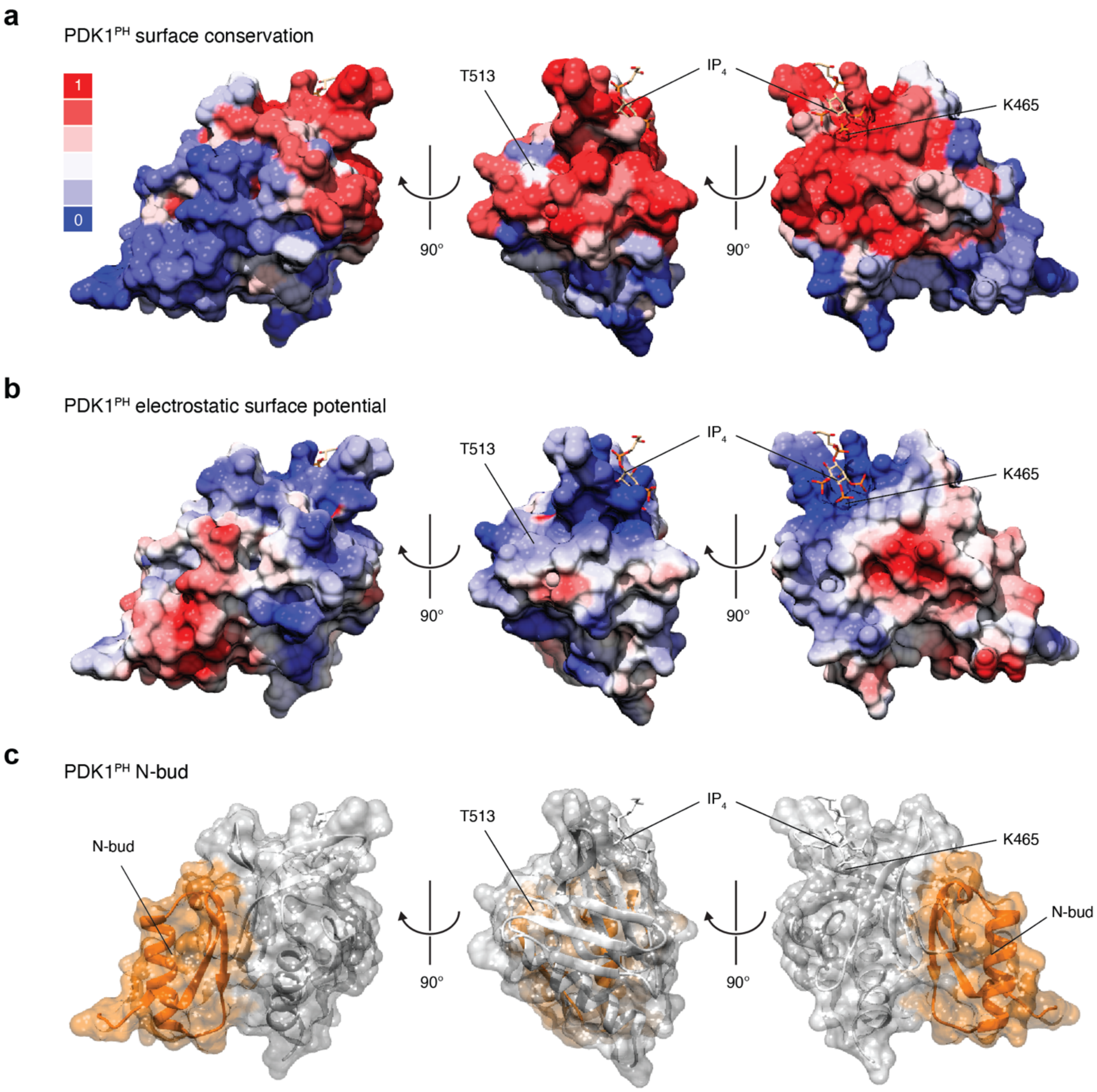

Supplementary Fig. 6. PDK1 is autoinhibited by its PH domain.

(A) PDK1 PH domain surface conservation mapped on the PH domain structure (PDB: 1w1d).

(B) PDK1 PH domain electrostatic surface potential mapped on the PH domain structure (PDB: 1w1d).

(C) PDK1 PH domain structure (PDB: 1w1d) with the N-bud highlighted in orange and annotated T513 and K465 residues.

Supplementary Fig. 7. PDK1 is autoinhibited by its PH domain.

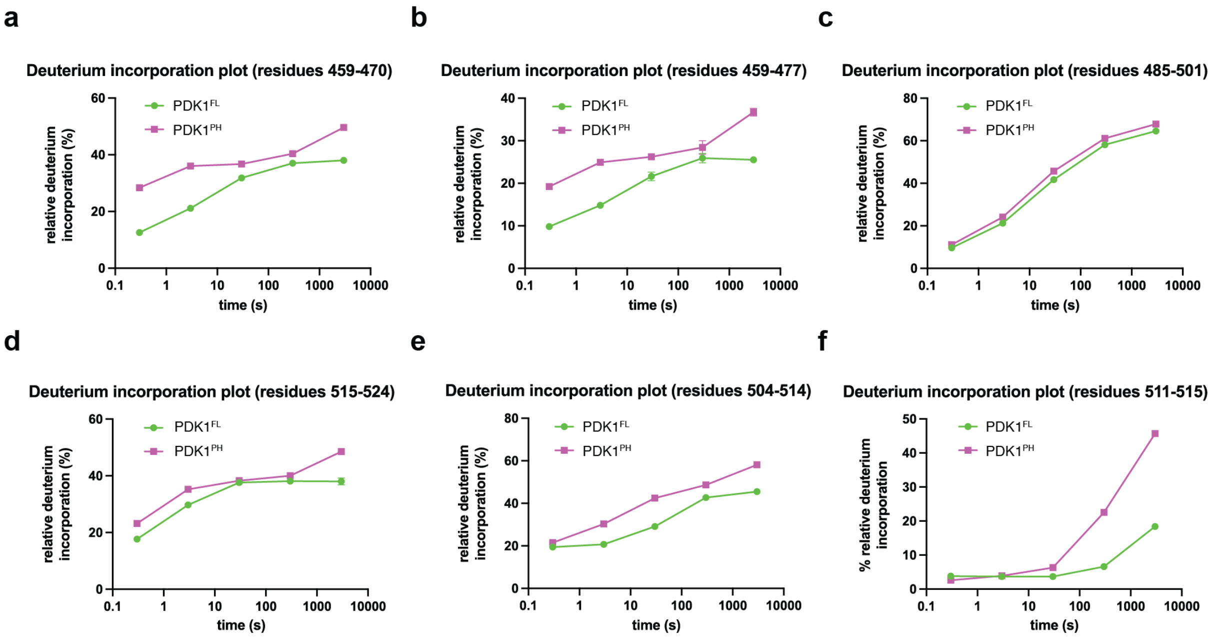

Supplementary Fig. 7. PDK1 is autoinhibited by its PH domain.

(A) – (C) Relative deuterium incorporation over time for peptides covering the PIP_3 binding site. n = 3 biologically independent experiments. Data are presented as mean values ± S.D.

(B) – (F) Relative deuterium incorporation over time for peptides covering the conserved surface that includes T513. n = 3 biologically independent experiments. Data are presented as mean values ± S.D.

Supplementary Fig. 8. PIP$_3$ drives cooperative, switch-like PDK1 activation.

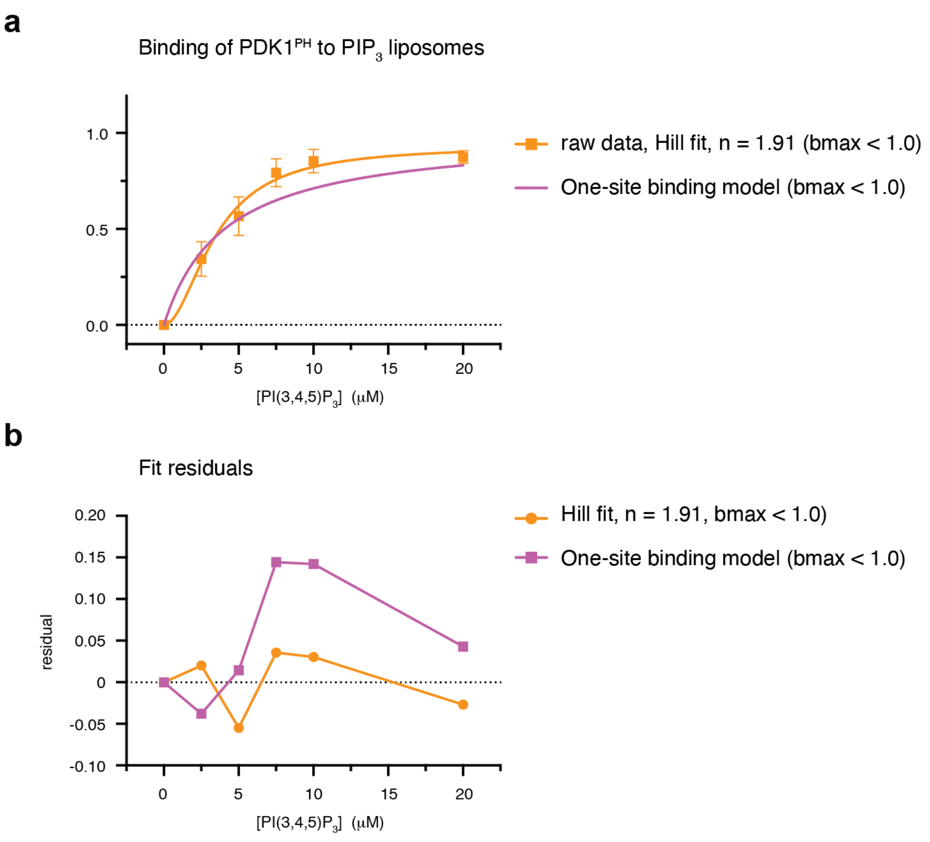

Supplementary Fig. 8. PIP$_3$ drives cooperative, switch-like PDK1 activation.

(A) Comparison of single-site and cooperative binding models derived from non-linear curve fitting for the binding of PDK1^{PH} to PIP$_3$-containing liposomes. n = 3 biologically independent experiments. Data are presented as mean values ± S.D.

(B) Residuals of the model fits depicted in A.

Supplementary Table 1. Constructs employed in this study.

Construct base	Nomenclature	Description of construct
PDK1 kinase domain (73-359)	PDK1^{SKD}	Wild-type short kinase domain
	PDK1^{SKD} S241A	Non-phosphorylatable variant
	PDK1^{SKD} Y288A	Dimer-disrupting mutant
	PDK1^{SKD} Y288E	Dimer-disrupting mutant
	PDK1$^{PIF-SKD}$	N-terminal PIFtide fusion (SKD)
	PDK1$^{SKD-PIF}$ S241A	C-terminal PIFtide fusion (SKD)
PDK1 long kinase domain (kinase domain + linker) (73-389)	PDK1^{LKD}	Wild-type long kinase domain
	PDK1^{LKD} S241A	Non-phosphorylatable variant
	PDK1^{LKD} F383A/M386A	HM mutant
	PDK1^{LKD} Y376A	'NFD' motif mutant
	PDK1$^{PIF-LKD}$	N-terminal PIFtide fusion (LKD)
PDK1 PH domain (408-556)	PDK1^{PH}	Wild type PH domain
PDK1 full-length (73-556)	PDK1^{FL}	Wild type full-length
	PDK1^{FL} S241A	Non-phosphorylatable variant

Supplementary Table 2. EDC cross-linking mass spectrometry of PDK1$^{SKD-PIF}$.

Cross-linked residues (PDK1$^{PIF-SKD}$)	Cross-linked peptide	Total peptide spectrum matches (PSM)	Dimer PSMs	Monomer PSMs
1-310	GPQPR(1)-DFDYIADW(7)	10	10	0
1-310	GPQPR(1)-LGSGGSSGSSGQEMFRDFDYIADW(23)	9	9	0
103-300	KIGSFDETCTR(1)-LGSGGSSGSSGQEMFR(13)	1	1	0
6-310	KKRPEDFK(1)-DFDYIADW(7)	1	1	0
6-306	KKRPEDFK(1)-DFDYIADW(3)	2	2	0
1-300	GPQPR(1)-LGSGGSSGSSGQEMFR(13)	9	9	0
7-306	KKRPEDFK(2)-DFDYIADW(3)	1	1	0
50-300	HIIKENK(4)-LGSGGSSGSSGQEMFR(13)	1	1	0
7-310	KKRPEDFK(2)-DFDYIADW(7)	2	2	0
	Total PSMs	36	36	0

Supplementary Table 3. EDC cross-linking mass spectrometry of $PDK1^{SKD-PIF}$ (activation loop and catalytic loop cross-linked peptides).

Cross-linked residues ($PDK1^{PIF-SKD}$)	Cross-linked peptide	Total peptide spectrum matches (PSM)	Dimer PSMs	Monomer PSMs
165-259	VLSPESKQAR(7)-LGCEEMEGYGPLK(5)	10	1	9
165-259	VLSPESKQAR(7)-RLGCEEMEGYGPLK(6)	2	0	2
165-258	VLSPESKQAR(7)-LGCEEMEGYGPLK(4)	3	0	3
165-186	VLSPESKQAR(7)-ANAFVGTAQYVSPELLTEK(18)	12	0	12
20-165	ILGEGSFSTVVLAR(4)-VLSPESKQAR(7)	5	0	5
165-217	VLSPESKQAR(7)-AGNEYLIFQK(4)	9	1	8
53-163	ENKVPYVTR(3)-VLSPESK(5)	3	0	3
165-182	VLSPESKQAR(7)-ANAFVGTAQYVSPELLTEK(14)	1	0	1
163-187	VLSPESK(5)-ANAFVGTAQYVSPELLTEKSACK(19)	2	0	2
[a]135-137	DLKPENILLNEDMHIQITDFGTAK(1)(3)	6	0	6
	Total PSMs	53	2	51

[a]Intra-catalytic loop cross-link.

Supplementary Table 4. BS^3 cross-linking mass spectrometry of $PDK1^{LKD}$.

**Cross-linked residues ($PDK1^{LKD}$)**	**Cross-linked peptide**	**Total peptide spectrum matches (PSM)**	**Dimer PSMs**	**Monomer PSMs**	**Model C_α-C_α distance (Å)**
76-120	KKRPEDFK(1)-HIIKENKVPYVTR(4)	11	1	10	26.6
76-120	KKRPEDFK(1)-HIIKENK(4)	13	0	13	26.6
77-120	KKRPEDFK(2)-HIIKENK(4)	8	0	8	26.2
83-120	RPEDFKFGK(6)-HIIKENKVPYVTR(4)	1	1	0	24.3
83-120	RPEDFKFGK(6)-HIIKENK(4)	2	2	0	24.3
76-173	KKRPEDFK(1)-KIGSFDETCTR(1)	7	7	0	41.6
83-173	RPEDFKFGK(6)-KIGSFDETCTR(1)	4	3	1	31.3
83-173	KKRPEDFKFGK(8)-KIGSFDETCTR(1)	2	2	0	31.3
120-173	HIIKENKVPYVTR(4)-KIGSFDETCTR(1)	9	9	0	15.7
120-173	HIIKENK(4)-KIGSFDETCTR(1)	4	3	1	15.7
	Total PSMs	61	27	33	

Supplementary Table 5. HDX-MS data processing.

**Data set**	**PDK1^{SKD} (monomer)**	**PDK1$^{SKD-PIF}$ (dimer)**	**PDK1^{FL}**	**PDK1^{SKD}**	**PDK1^{FL}**	**PDK1^{PH}**
HDX reaction details	%D_2O=66%<br>pH $_{(read)}$=7.5<br>Temp=18˚C	%D_2O=66%<br>pH $_{(read)}$=7.5<br>Temp=18˚C	%D_2O=89.6%<br>pH $_{(read)}$=7.5<br>Temp=18˚C	%D_2O=89.6%<br>pH $_{(read)}$=7.5<br>Temp=18˚C	%D_2O=81.2%<br>pH $_{(read)}$=7.5<br>Temp=18˚C	%D_2O=81.2%<br>pH $_{(read)}$=7.5<br>Temp=18˚C
HDX time course (seconds)	3, 30, 300, 3000	3, 30, 300, 3000	3s at 4˚C, 3, 30, 300, 3000	3s at 4˚C, 3, 30, 300, 3000	3s at 4˚C, 3, 30, 300, 3000	3s at 4˚C, 3, 30, 300, 3000
HDX controls	N/A	N/A	N/A	N/A	N/A	N/A
Back-exchange	No correction	No correction	No correction	No correction	No correction	No correction
Number of peptides	46	46	102	102	69	69
Sequence coverage	96.9%	96.9%	93.1%	93.1%	94.8%	94.8%
Average peptide length/<br>redundancy	Length=15.0<br>Redundancy=<br>2.4	Length=15.0<br>Redundancy=<br>2.4	Length=13.0<br>Redundancy=<br>4.6	Length=13.0<br>Redundancy=<br>4.6	Length=18.4<br>Redundancy=<br>8.3	Length=18.4<br>Redundancy=<br>8.3
Replicates	3	3	3	3	3 (2 for 3000s)	3 (2 for 30s)
Repeatability	Average StDev=0.6%	Average StDev=0.5%	Average StDev=0.6%	Average StDev=0.5%	Average StDev=0.6%	Average StDev=0.7%
Significant differences in HDX	≥5% and ≥0.4 Da and unpaired t-test ≤0.01	≥5% and ≥0.4 Da and unpaired t-test ≤0.01	≥5% and ≥0.4 Da and unpaired t-test ≤0.01	≥5% and ≥0.4 Da and unpaired t-test ≤0.01	≥5% and ≥0.4 Da and unpaired t-test ≤0.01	≥5% and ≥0.4 Da and unpaired t-test ≤0.01
